# Gene expression profiling of inflammatory cytokines in esophageal biopsies of different phenotypes of gastroesophageal reflux disease: a cross-sectional study

**DOI:** 10.1186/s12876-021-01707-7

**Published:** 2021-05-03

**Authors:** Mónica R. Zavala-Solares, Gabriela Fonseca-Camarillo, Miguel Valdovinos, Julio Granados, Guido Grajales-Figueroa, Luis Zamora-Nava, Nancy Aguilar-Olivos, Luis R. Valdovinos-García, Jesús K. Yamamoto-Furusho

**Affiliations:** 1 Programa de Doctorado en Ciencias Médicas, Unidad de Posgrado, Universidad Nacional Autónoma de México, Mexico City, Mexico; 2 Inflammatory Bowel Disease Clinic, Department of Gastroenterology, Instituto Nacional de Ciencias Médicas y Nutrición Salvador Zubirán, Vasco de Quiroga #15. Col. Sección XVI, CP 14000 México City, D.F. Mexico; 3 GI Motility and Neurogastroenterology Unit, Departament of Gastroenterology, Instituto Nacional de Ciencias Médicas y Nutrición Salvador Zubirán, Vasco de Quiroga #15. Col. Sección XVI, México City, Mexico; 4 Department of Transplantation, Instituto Nacional de Ciencias Médicas y Nutrición Salvador Zubirán, Mexico City, Mexico; 5 Department of Endoscopy, Instituto Nacional de Ciencias Médicas y Nutrición, Salvador Zubirán, Mexico City, Mexico

**Keywords:** Esophagitis, Non-Erosive Reflux Disease (NERD), Cytokine, Gene

## Abstract

**Background:**

The clinical endoscopic phenotypes of gastroesophageal reflux disease (GERD) are classified as Barrett's esophagus (BE), erosive esophagitis (EE) and non-erosive gastroesophageal reflux disease (NERD). NERD is subclassified as abnormal acid exposure (AAE) and normal acid exposure (NAE) based on pH monitoring study results. The aim of this study was to characterize genes involved in the pathophysiology and immune response of GERD.

**Methods:**

This is an observational and cross-sectional study. All patients with BE, EE, AAE, and NAE and a control group were subjected to superior endoscopy (with biopsies of esophageal mucosa). Relative mRNA quantification of cytokine and target genes was conducted by quantitative Polymerase Chain Reaction (RT-qPCR). Changes in the expression of genes associated with inflammation were assessed for each disease phenotype. Statistical analysis of differential gene expression was performed using the Mann–Whitney U non-parametric test. A *p* value < 0.05 was considered significant.

**Results:**

A total of 82 patients were included and were divided into the following groups: Group BE, 16 (19.51%); Group EE, 23 (28.04%); Group AAE, 13 (15.86%); NAE 13 (15.86%); and Control Group, 17 (20.73%). Compared with the control group, patients with BE exhibited increased IL-8 expression (*p* < 0.05) and increased levels of IL-10, MMP-3, and MMP-9. Patients with EE exhibited increased levels of IL-1B, IL-6 and IL-10 (*p* < 0.05), and patients with AAE exhibited increased expression of IL-1B, IL-6, IFN-γ and TNF-α (*p* < 0.05). AAE exhibited increased IL-1B and TNF-α expression compared with NAE (*p* < 0.05).

**Conclusion:**

This study demonstrates the differential expression of mediators of inflammation in the esophageal mucosa of patients with different GERD endoscopic phenotypes. IL-1B and TNF-α could be useful to differentially diagnose AAE and NAE in the non-erosive phenotype using endoscopic biopsies.

## Background

Gastroesophageal reflux disease (GERD) is a multifactorial disease, and it is one of the most frequent pathologies in the outpatient clinic of gastroenterology. GERD is defined as the presence of heartburn and regurgitation 1 to 2 times per week for at least one month. GERD requires increased esophageal exposure to gastric content. The pathophysiology of GERD is complex, involving mechanical factors, such as the presence of hiatal hernia and transient relaxations of the lower esophageal sphincter [1].

Patients are classified into the following clinical categories based on their clinical endoscopic phenotype: Barrett's esophagus (BE), erosive esophagitis (EE) and non-erosive gastroesophageal reflux disease (NERD). The NERD group is divided into abnormal acid exposure (AAE) and normal acid exposure (NAE) according to the esophageal exposure time at pH < 4 based on pH monitoring studies.

The severity of GERD injuries and symptoms cannot be predicted exclusively based on esophageal exposure [2]. Patients display distinctive clinical presentation. Some may have erosions, whereas others exhibit a non-erosive disease that suggests that other factors may be involved [3].

The expression of cytokines (both inflammatory and anti-inflammatory) in the esophageal mucosa in patients with GERD symptoms and their endoscopic phenotypes have been evaluated in different studies. Previous studies of GERD biopsies in humans have demonstrated increased expression of IL-1B, TNF-α, IL-8 and IL-10 [4–6]. Some studies did not cover all the phenotypes of the disease, and those studies that included the non-erosive phenotype did not include the pH monitoring study within their methodology to distinguish those patients with normal esophageal exposure to acid from those without.

Studies performed in a mouse model of gastroesophageal reflux showed upregulation of inflammatory-related genes, especially NF-κB target genes (matrix metalloproteinases-3 and -9, IL-1β, IL-6, and IL-8) [3]. To our knowledge, these matrix metalloproteinases have not been studied in GERD in humans.

Recently Bonfiglio et al. [7] provided evidence for 30 independent loci that are involved in molecular pathways with biological relevance to the pathophysiology of GERD. This study proposed initial insights into the genetic background of GERD, which was further supported by genome wide association study (GWAS) analyses that showed that GERD, Barrett’s esophagus and esophageal adenocarcinoma exhibit substantial overlap in terms of genetic etiology. Despite being one of the most frequent pathologies in gastroenterology, there are still gaps in knowledge on pathophysiology to explain why a patient presents a certain phenotype. Genetics could play a role in explaining the presence of lesions in certain phenotypes.

Advances in discovery of new pathways involved in the etiopathogenesis of GERD highlight the crucial role of regulation of local inflammatory responses.

The aim of the present study was to characterize critical genes involved in the immune response of GERD in each endoscopic phenotype and to assess potential differences in gene expression in the non-erosive endoscopic phenotype based on esophageal acid exposure time.

## Methods

### Selection of GERD patients

This is a cross-sectional study of patients with typical symptoms of gastroesophageal reflux disease (heartburn, regurgitation). This research was performed at the Department of Gastroenterology and Endoscopy at Instituto Nacional de Ciencias Médicas y Nutrición Salvador Zubirán (INCMNSZ). All patients who agreed to participate were subjected to superior endoscopy (already referred by their treating physician for this study) and were classified into 3 groups based on the endoscopy findings: BE (short segment < 3 cm, long segment > 3 cm), EE (Los Angeles Classification) and NERD. The last group was subclassified into abnormal acid exposure (AAE) or normal acid exposure (NAE) according to 24-h pH monitoring study results.

Selection criteria for patients with GERD included typical symptoms (heartburn and/or regurgitation) at least once a week for at least 1 year. Subjects were older than 18 years of age and included both genders. All of the subjects agreed to participate in the study by signing a consent form. The control group included patients with dyspepsia who at the time of their endoscopy presented a macroscopically gastric mucosa without lesions. The pH monitoring study results for control patients were subsequently negative for abnormal esophageal exposure to acid reflux.

All groups underwent biopsies of esophageal mucosa during the endoscopic procedure. If lesions were found (BE, EE), two biopsies were obtained: one from the injured region (BE, EE) and another from the adjacent mucosa without injury that was 5 cm above the esophageal mucosal junction when it was free of injured mucosa. If no lesions were found, a biopsy of the esophageal mucosa was obtained 5 cm above the esophageal mucosal junction (one biopsy for the non-erosive endoscopic phenotype and control group).

Prior to endoscopy, patients who did not present erosions underwent a pH monitoring study for classification according to the level of acid exposure in the esophagus. A 24-h esophageal pH monitoring study with a 1-sensor catheter (GeroFlex, Alpine Biomed, Fountain Valley CA, EU) placed 5 cm above the lower esophageal sphincter, which was located by esophageal manometry, was performed. A portable recording device (Digitrapper, Medtronic, Parkway, Minneapolis, MN, USA) was used. The pH monitoring study was performed when the patient was off proton pump inhibitors. These patients were subsequently subclassified with esophageal abnormal acid exposure (AAE) (presence of abnormal acid reflux) and esophageal normal acid exposure (NAE). Symptom analysis was not considered for the purposes of this study. According to the results of the percentage of exposure time at pH < 4, patients were classified as AAE (exposure time percentage > 4.2%) or NAE (percentage of exposure time < 4.2%).

### Operational definitions

BE: Patients with long segment (> 3 cm) and short segments (< 3 cm) of the epithelial column located between the upper border of the gastric folds and the proximal part of the Z line and the presence of intestinal metaplasia was histopathologically confirmed in biopsies of the Barrett epithelium segment.

EE: Patients with GERD symptoms with erosions or disruptions of the esophageal mucosa of different degrees by Los Angeles Classification as follows: Grade A, one (or more) mucosal break less than 5 mm that does not extend between the tops of two mucosal folds; Grade B, one (or more) mucosal break greater than 5 mm long that does not extend between the tops of two mucosal folds; Grade C, one (or more) mucosal break that is continuous between the tops of two or more mucosal folds but involves less than 75% of the circumference; and Grade D, one (or more) mucosal break that involves at least 75% of esophageal circumference [8].

Non-erosive phenotype: GERD symptoms but no lesions identified by endoscopy.

Abnormal acid exposure (AAE): GERD symptoms, no lesions identified by endoscopy and a pH monitoring study with > 4.2% exposure time at pH < 4.

Normal acid exposure (NAE): GERD symptoms, no lesions at endoscopy and a pH monitoring study with < 4.2% exposure time at pH < 4.

Control group (C): Patients without pathology involving their immunity (neoplasms, celiac disease, rheumatic diseases) who present dyspepsia under study with normal endoscopy (without organic disease) and with normal pH monitoring study results (which excludes gastroesophageal reflux).

### Sample processing and gene expression analysis

Based on previous studies, the expression of the following cytokines and inflammation mediators were analyzed: IL1B, IL-6, IL-8, IL-10, TNF-α, IFN-γ, MMP-3 and MMP-9.

The esophageal mucosal biopsies obtained from endoscopy were immediately placed in RNA later (Ambion, Austin, TX, USA) and stored at -70 °C (short-term; < 6 months) until use. Then, total RNA was isolated using High Pure RNA Tissue (Roche Diagnostics, Mannheim, Germany) following the manufacturer’s guidelines. Two hundred nanograms of total RNA was reverse transcribed into cDNA with random hexamer primers (Roche Diagnostics, Mannheim, Germany). The methodology employed was based on the previous studies of gene expression [9–11]. RT-qPCR amplification was performed with 20 ng of cDNA, 200 nM forward and reverse primers, and Taqman Master Mix (Roche Diagnostics, Mannheim, Germany Roche Diagnostics, Mannheim, Germany) in a final volume of 10 µl (Table 1). PCR reactions were run in a Light Cycler 480 (Roche Diagnostics, Mannheim, Germany) for 45 cycles. Each cycle consisted of denaturation for 15 s at 95°, primer annealing for 15 s at 55 °C, extension for 30 s at 72 °C and cooling 30 s at 40 °C.

**Table 1:** Primers designs from universal probe library

Gene	Genebank	Oligonucleotides	Probe UPL
IL-1β	NM_000576.2	tacctgtcctgcgtgttgaa tctttgggtaatttttgggatct	♯78
IL-6	NM_000600.3	gcccagctatgaactccttct cttctcctgggggtactgg	♯ 68
IL-8	NM_000584.2	agacagcagagcacacaagc atggttccttccggtggt	♯72
IL-10	NM_000572.2	cataaattagaggtctccaaaatcg aaggggctgggtcagctat	♯45
IFN-γ	NM_000619.2	ggcattttgaagaattggaaag tttggatgctctggtcatctt	♯21
TNF-α	NM_000594.2	cgctccccaagaagacag agaggctgaggaacaagcac	♯57
MMP3	NM_002422.3	caaaacatatttctttgtagaggacaa ttcagctatttgcttgggaaa	♯36
MMP9	NM_004994.2	gaaccaatctcaccgacagg gccacccgagtgtaaccata	♯6
GAPDH	NM_002046.3	agccacatcgctcagacac gcccaatacgaccaaatcc	♯60

UPL (Universal Probe Library)

To ensure quality control of RT-qPCR assays, linearity and reproducibility were determined (VC < 10%). The relative quantification of mRNA of target genes was conducted using the LightCycler software 4.1 according to the 2-delta Ct method.

Glyceraldehyde 3-phosphate dehydrogenase (GAPDH) mRNA levels were used to standardize esophageal tissues from patients with disease and samples without inflammation.

Changes in gene expression were assessed and represented by relative gene expression units of target/housekeeping gene in each disease phenotype. The following inflammatory molecules were assessed and compared with the control group: IL-1B, IL-6, IL-8, IL-10, TNF-α, IFN-γ, MMP-3, MMP-9.

Real-time PCR and RT-qPCR reactions differ from a regular PCR reaction since a fluorophore is released during the amplification, the amount of which correlates to the amount of template copies created. Since the number of copies theoretically doubles in each cycle, when comparing two amplifications, the assay with more copies of a template in a sample will amplify faster and release quantifiable fluorescence in an early cycle [12].

### Statistical analysis

Acid exposure times in each subgroup of the non-erosive phenotype and controls were compared using the Kruskal–Wallis test.

Patients with lesions (EE and BE) had 2 biopsies: one biopsy of the lesion and another biopsy of healthy mucosa. Expression of each gene was analyzed using the Wilcoxon's test, and each patient served as his/her own control. For analysis purposes, biopsies of the lesion tissue (EE, BE) were used for comparisons with the control group.

Statistical analysis of differential gene expression was performed using the Mann–Whitney U non-parametric test. A *p* value < 0.05 was considered significant. Analysis was performed using (Statistical Package for Social Sciences) SPSS version 20 and Prism GraphPad version 6.

## Results

### Demographic and clinical characteristics

Out of 82 included patients, 64.6% were women with a median age of 59 years. Patients were divided into the following groups: Group BE, 16 (19.51%); Group EE, 23 (28.04%); Group AAE, 13 (15.86%); NAE, 13 (15.86%); and Control Group, 17 (20.73%). Demographic characteristics based on group, endoscopic findings and acid exposure time are detailed in Table 2.

**Table 2:** Demographic characteristics of patients with GERD and Controls, endoscopic findings and Acid exposure time in 24 h

	EE	BE	AAE	NAE	Control
n (%)	23 (28.04%)	16 (19.51%)	13 (15.85)	13 (15.85)	17 (20.73)
Age years median(IQ)	50 (34–60)	56 (42.64)	49 (40–54)	31 (25–43)	45 (32–52)
Gender					
Female n (%)	12 (52.18)	9 (56.25)	9 (69.24)	10 (76.92)	13 (76.48)
Male N (%)	11 (47.82)	7 (43.75)	4 (30.76)	3 (23.07)	4 (23.52)
Endoscopic Findings n	Esofagitis A–B: 16 C–D: 7	Segment < 3 cm: 10 > 3 cm: 6	Non erosive	Non erosive	Non erosive
Acid exposure time in 24 h median% (IQ)	NA	NA	13.65 (8.55–20.1)	1.0 (0.2–1.6)	0.6 (0–1.1)

*EE* Esophageal esophagitis, *BE* Barrett’s esophagus, *AAE* Abnormal acid exposure, *NAE* normal acid exposure, *F* Female, *NA* not available

The median (IQ) of the acid exposure time was obtained during the pH monitoring study of each subgroup of the non-erosive endoscopic phenotype (Table 2). Comparing the percentage obtained between the groups, significant differences (0.001) were observed with a higher percentage in the AAE subgroup as expected. The NAE group and controls were specifically compared. Although the median percentage was increased in the NAE subgroup, no statistically significant differences were noted between these two groups.

### Differential gene expression of pro-inflammatory mediators in GERD patients.

**Fig. 1:**
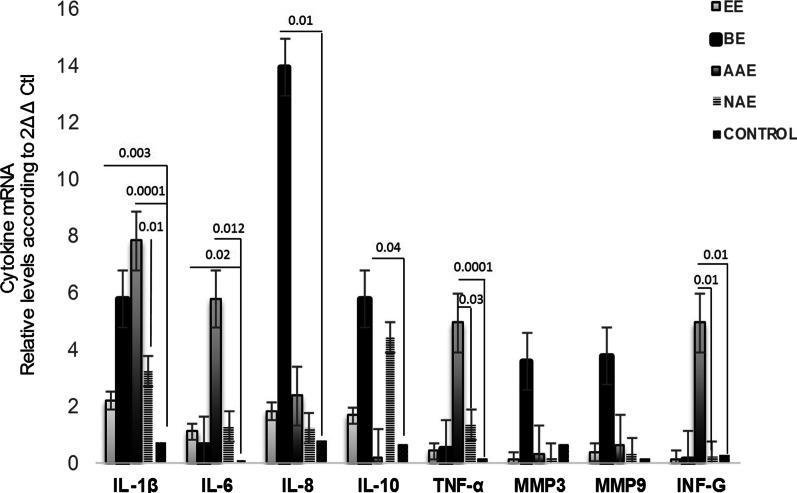
Global comparison of gene expression profile of all cytokines in injured mucosa of Barrett’s esophagus (BE), Erosive esophagitis (EE), Abnormal acid exposure (AAE), Normal acid exposure (NAE) and controls (C) mRNA levels. Bars show mean ± SEM of the mean of transcript levels from patients with GAPDH as housekeeping gene determined by 2-∆Ct * *p* < 0.05

**Fig. 2:**
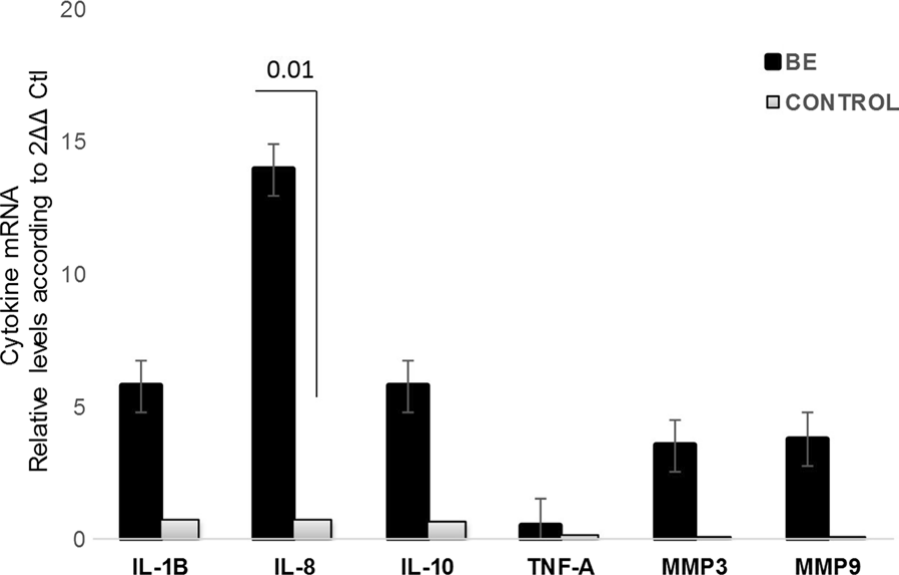
Gene expression profile in injured mucosa of Barrett’s esophagus (BE) and controls (C) mRNA levels. Bars show mean ± SEM of the mean of transcript levels from BE patients with GAPDH as housekeeping gene determined by 2-∆Ct * *p* < 0.05

**Fig. 3:**
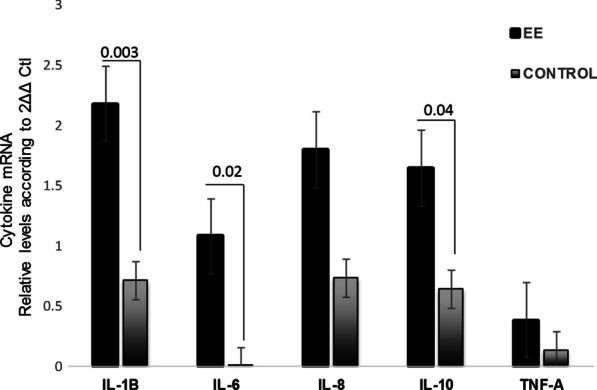
Gene expression profile in injured mucosa of Erosive esophagitis (EE) and controls (C) mRNA levels. Bars show mean ± SEM of the mean of transcript from EE patients with GAPDH as housekeeping gene determined by 2-∆Ct * *p* < 0.05

**Fig. 4:**
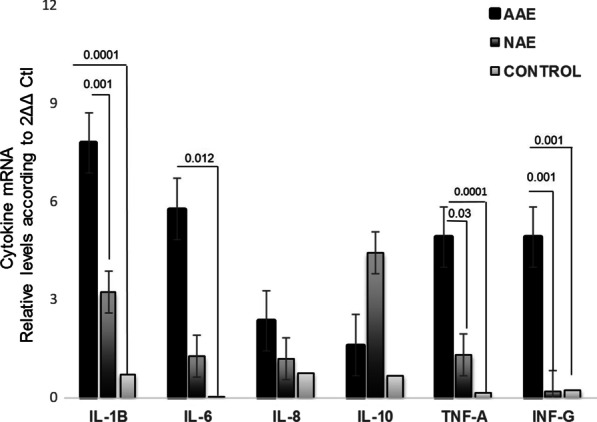
Gene expression profile of inflammatory cytokines with Abnormal acid exposure (AAE), Normal acid exposure (NAE) and controls (C) mRNA levels. Bars show mean ± SEM of the mean of transcript levels in colonic mucosa from NERD patients with GAPDH as housekeeping gene determined by 2-∆Ct * *p* < 0.05

Relative gene expression of pro-inflammatory cytokines was detectable and quantifiable by RT-qPCR in biopsies of different phenotypes of gastroesophageal reflux disease and controls. Figure 1 presents a global comparison of relative gene expression of pro-inflammatory cytokines in all the phenotype groups and the control group. Comparing the expression of genes in all groups, BE stands out with a predominance of IL-8, IL-10, MMP-3 and MMP-9 expression, and IL-1B, INF-γ, IL-6 and TNF-α expression is notable in the AAE group. Figures 2, 3 and 4 correspond to the comparisons of each of the phenotypes with the control group.

No differences were noted when comparing the biopsies of lesion versus healthy mucosa in the same patient in the BE and EE groups; therefore, it was decided that all comparisons of the EE and BE groups were analyzed using the injured tissue samples. No differences were found when comparing patients with BE > 3 cm and < 3 cm.

### Gene expression profile in patients with Barrett’s esophagus and controls

Patients with BE exhibited increased IL-8 expression compared with the control group (*p* = 0.010) (Fig. 2). Also, we detected higher levels of inflammatory mediators, such as IL-1B, MMP-3 and MMP-9, in samples of patients with BE compared with the control group.

### Gene expression profile in patients with erosive esophagitis and controls

Patients with EE exhibited significantly higher levels of relative expression of IL-1B, IL-6 and IL-10 mRNA compared to controls (*p* = 0.003, *p* = 0.02 and *p* = 0.04, respectively) (Fig. 3). Multiple comparisons corroborated a significant predominance of IL-1B compared with the remaining cytokines.

### Gene expression profile in patients with the non-erosive endoscopic phenotype subclassified as AAE/NAE and controls

Patients with non-erosive endoscopic phenotype were subclassified as AAE/NAE Fig. 4. Patients with AAE exhibited increased expression of IL-1B, IL-6 and TNF-α compared to the control group (*p* < 0.05). Patients in the AAE group exhibited increased IL-1B and TNF-α expression compared with the NAE group (*p* < 0.05). Relative gene expression of pro-inflammatory cytokines in biopsies of different phenotypes of gastroesophageal reflux disease and controls are depicted in Table 3.

**Table 3:** Gene expression of inflammatory cytokines in each of the gastro-esophageal reflux disease phenotypes compared with the control group and in the non-erosive subgroup

	IL-1β Mean ± SEM *p* value	IL-6 Mean ± SEM *p* value	IL-8 Mean ± SEM *p* value	IL-10 Mean ± SEM *p* value	INF-γ Mean ± SEM *p* value	TNF-α Mean ± SEM *p* value	MMP3 Mean ± SEM *p* value	MMP9 Mean ± SEM *p* value
Barrett’s Esophagus versus Control	0.057 ± 0.050 N.S	0.002 ± 0.005 N.S	0.24 ± 0.01 0.010	0.024 ± 0.025 N.S	0.05 ± 0.04 N.S	0.04 ± 0.03 N.S	0.018 ± 0.033 N.S	0.023 ± 0.04 N.S
Erosive esophagitisversus Control	0.46 ± 0.031 0.003	0.03 ± 0.002 0.020	0.010 ± .007 N.S	0.010 ± 0.009 0.043	0.001 ± 0.003 N.S	0.002 ± 0.001 N.S	0.003 ± 0.002 N.S	0.003 ± 0.002 N.S
Abnormal acid exposure versus Control	0.19 ± 0.10 0.0001	0.057 ± 0.047 0.012	0.023 ± 0.017 N.S	0.01 ± 0.01 N.S	0.018 ± 0.013 0.001	0.049 ± 0.028 0.0001	0.009 ± 0.007 N.S	0.005 ± 0.001 N.S
Abnormal acid exposure versus Normal Acid Exposure	0.16 ± 0.10 0.001	0.04 ± 0.05 N.S	0.011 ± 0.023 N.S	0.001 ± 0.003 N.S	0.004 ± 0.014 N.S	0.03 ± 0.030 0.036	0.03 ± 0.09 N.S	0.006 ± 0.005 N.S

Error of the mean (SEM), No Significative (NS)

## Discussion

In the present study, we assessed the expression of cytokines involved in the pathophysiology and immune response of GERD. This study analyzed gene expression of inflammatory mediators (IL-1B, IL-6, IL-8, IL-10, INF-γ and TNF-α,) and two metalloproteases associated with esophageal mucosa damage as reported in previous studies in patients with 3 different endoscopic phenotypes of the disease (BE, EE and NERD). Gene expression was also compared in patients with the non-erosive variety who underwent pH monitoring for 24 h to differentiate cases with abnormal esophageal acid exposure from those with normal exposure.

Patients with BE exhibited increased expression of IL-8, IL-10, MMP-9 and MMP-3; patients with EE demonstrated increased levels of IL-1B, IL-6 and IL-10. AAE stood out with higher levels of IL-1B, INF-γ, IL-6 and TNF-α. AAE exhibited increased expression of IL-1B and TNF-α compared with NAE. A relevant difference in the groups with true pathological reflux and those without metaplasia (EE and AAE) is increased expression in IL-10 in EE compared to AAE.

Although this is a descriptive study, the findings are of interest. To the best of our knowledge, this is the first study to analyze all endoscopic phenotypes of patients with typical GERD symptoms, assess the non-erosive endoscopic phenotype using a pH monitoring study, and characterize gene expression in these patients according to pH monitoring study results.

The profile of mediators in BE provide further understanding of the pathogenesis of Barrett's esophagus and the possible immunoregulatory role of IL-10 associated with the permanent mucosal damage that is unable to counteract the aggression of other inflammation mediators. This finding is in accordance with a previous study reported using a mouse model of Barrett’s esophagus that showed increased expression of IL-10 compared with mice with non-Barrett's esophagus; however, no differences in the levels of pro-inflammatory cytokines, such as TNF-α or INF-γ, were noted [13].

On the other hand, MMP3 and MMP9 expression was increased in BE compared to the control group, and the expression of these genes predominate in this phenotype. We found that the expression of this protease was increased although our group of patients with BE did not exhibit dysplasia [14]. Further studies are required to determine if metalloproteins play a role in mucosal damage in BE. Our study highlights the participation of MMP-9 and MMP-3 in patients with BE, which possibly explains the pathological mechanism for this epithelial change.

Regarding IL-8 measurements, we observed increased expression of this cytokine in patients with BE compared to controls. Chronic inflammation in BE may play a critical role in the progression from benign to malignant esophageal disease [15].

On the other hand, the erosive phenotype is a classic example of the balance that exists between the expression of Th1 and Th2 in response to the aggression presented (reflux). In this study, we demonstrated increased expression of pro-inflammatory cytokines, such as IL-1B and IL-6, in mucosal biopsies of patients with EE. Other authors have presented similar findings in patients with the erosive variety [4, 16–18].

Interestingly, we found an increased IL-10 gene expression in mucosa of patients with EE compared with controls, and these results suggest the possible role of IL-10 as a critical cytokine in the immunoregulatory mechanism in the inflammatory chronic response in the esophagus. IL-10 is a cytokine with potent anti-inflammatory properties that plays a central role in limiting host immune response, thereby preventing damage to the host and maintaining normal tissue homeostasis. Dysregulation of IL-10 is associated with an enhanced immunopathology response to damage. Thus, a fundamental understanding of IL-10 gene expression is critical for our comprehension of disease progression and resolution of the host inflammatory response.

Recent studies have provided greater insight into the pathophysiology and symptom generation in NERD [19].

In patients with the non-erosive phenotype, this study allowed us to characterize patients with AAE and NAE. The AAE subgroup, which was confirmed to exhibit pathological reflux as corroborated by the pH monitoring study, exhibited similarities with the EE group, namely increased expression of IL-1B and IL-6. These genes could be two markers in patients with real GERD who do not present metaplasia. AAE also presented unique differences compared with all groups, such as significant increases in INF-γ and TNF-α compared with the control group. The expression of NAE cytokines in AAE was very similar to that noted in the control group. One finding that highlights the differences between AAE and NAE is the increased expression of IL-1B and TNF-α in AAE. In a subsequent study of diagnostic accuracy, it would be worth determining whether IL-1B/TNF-α represent useful biomarkers in biopsies at the time of endoscopy to define AAE/NAE for non-erosive endoscopic phenotypes. Interestingly, we found decreased expression of IL-10 in patients with AAE and NAE compared with controls.

In this study, we revealed a similar expression profile between EE and AAE with increased expression of IL-1B and IL-6. However, even these findings do not explain why patients with pathological reflux can present erosions and others do not (despite the fact that the median of patients with % time with pH < 4 was 13.65%, which is threefold more than the cutoff point). When comparing the expression of cytokines in these two groups, this finding potentially indicated that these cytokines are involved in the mechanism of mucosal repair and damage. Further investigation is necessary to corroborate these findings.

As noted above, we found that IL-8 is mainly increased in BE. IL-8 expression is not part of the significant findings in EE or AAE. Contrary to our results, some authors have reported an increase in IL-8 in NERD patients. Previously, Kanazawa et al. [20] detected increased IL-8 expression in NERD compared to asymptomatic subjects not subject to pH monitoring studies. Yoshida [21] also reported an increase the expression of this chemokine in NERD patients.

This study reports gene expression profiling of inflammatory mediators in the esophagus tissue from patients with different GERD phenotypes.

Studying the molecular and genetic bases of a disease is of fundamental importance as this information it offers in-depth insight into its pathogenesis and opens new routes for diagnosis and specific treatment. Understanding this pathway could lead us to the possibility of reversing the alteration of these genes and distinguishing these genes from those that are involved in erosions or lesions in GERD patients compared to the NERD phenotype.

## Conclusion

This study demonstrated the differential expression of mediators of inflammation in the esophageal mucosa of patients with different endoscopic phenotypes. IL-1B and TNF-α could be useful to differentially diagnose AAE and NAE in the non-erosive phenotype using endoscopic biopsies. The mucosal damage pathways could be mediated by MMP3 given that mucosal rupture is induced by the family of metalloproteins in BE. Further studies are needed to corroborate remission of these findings with medical or surgical treatments for GERD.

## Data Availability

All data used to support the findings of this study are included within the article in Tables 1, 2, 3 and Figs. 1, 2, 3 and 4. There are no supplementary data files attached.

## Abbreviations

BE: Barrett's esophagus
cDNA: Complementary DNA
ECM: Extracellular matrix
EE: Erosive esophagitis
GAPDH: Glyceraldehyde-3-phosphate dehydrogenase
GWAS: Genome-wide association study
IFN-γ: Interferon γ
IL-10: Interleukin 10
IL-1B: Interleukin 1B
IL-6: Interleukin 6
IL-8: Interleukin 8
MMP3: Matrix metalloproteinase 3
MMP9: Matrix metalloproteinase 9
NERD: Non-erosive gastroesophageal reflux disease
RNA: Ribonucleic acid
RT-PCR: Real Time Polymerase Chain Reaction
SEM: Standard error of the mean
TNF-α: Tumor necrosis factor α
